# A new look at the drug-resistance investigation of uropathogenic *E. coli* strains

**DOI:** 10.1007/s11033-017-4099-y

**Published:** 2017-01-13

**Authors:** Wioletta Adamus-Białek, Łukasz Lechowicz, Anna B. Kubiak-Szeligowska, Monika Wawszczak, Ewelina Kamińska, Magdalena Chrapek

**Affiliations:** 1 Institute of Medical Sciences, Jan Kochanowski University, IX Wieków Kielc 19A Av., 25-317 Kielce, Poland; 2 Department of Microbiology, Institute of Biology, Jan Kochanowski University, 15 Swietokrzyska St., 25-406 Kielce, Poland; 3 Institute of Mathematics, Jan Kochanowski University, 15 Swietokrzyska St., 25-406 Kielce, Poland; 4 Institute of Medical Biology, Polish Academy of Sciences, 106 Lodowa St., 93-232 Lodz, Poland

**Keywords:** UPEC, Drug resistance, Differentiation, ATR/FT-IR

## Abstract

Bacterial drug resistance and uropathogenic tract infections are among the most important issues of current medicine. Uropathogenic *Escherichia coli* strains are the primary factor of this issue. This article is the continuation of the previous study, where we used Kohonen relations to predict the direction of drug resistance. The characterized collection of uropathogenic *E. coli* strains was used for microbiological (the disc diffusion method for antimicrobial susceptibility testing), chemical (ATR/FT-IR) and mathematical (artificial neural networks, Ward’s hierarchical clustering method, the analysis of distributions of inhibition zone diameters for antibiotics, Cohen’s kappa measure of agreement) analysis. This study presents other potential tools for the epidemiological differentiation of *E. coli* strains. It is noteworthy that ATR/FT-IR technique has turned out to be useful for the quick and simple identification of MDR strains. Also, diameter zones of resistance of this *E. coli* population were compared to the population of *E. coli* strains published by EUCAST. We observed the bacterial behaviors toward particular antibiotics in comparison to EUCAST bacterial collections. Additionally, we used Cohen’s kappa to show which antibiotics from the same class are closely related to each other and which are not. The presented associations between antibiotics may be helpful in selecting the proper therapy directions. Here we present an adaptation of interdisciplinary studies of drug resistance of *E. coli* strains for epidemiological and clinical investigations. The obtained results may be some indication in deciding on antibiotic therapy.

## Introduction

The observed increase of drug resistance among bacteria forces to seek still new solutions to this problem [1, 2]. This is also an interesting issue for studying the mechanisms and correlations between bacteria, antibiotics and the host [3]. A very useful tool for data analysis is mathematical modeling which is increasingly used in biology, with particular success in epidemiology [4, 5]. This method uses theoretical assumptions and mathematical tools for assessing the dynamics between antibiotic consumption and the occurrence of antimicrobial resistance [6]. Mathematical modeling allows to analyze complex relationships among interdependent parameters [7]. There are many factors that influence the acquisition of resistance in bacteria and modulate antibiotic resistance patterns. The significance of individual factors is difficult to determine and the mathematical analysis may be helpful here. The value of these factors may be crucial for the investigation of changes in antibiotic resistance [4]. Mathematical modeling is appropriable to the study of dynamics of this phenomenon and predicts its development [6, 8]. Another use of modeling is guiding clinical practice. For example, this method may be used to determine the appropriate antibiotic therapy to prevent the induction of resistance [1, 9]. The combination therapy with two antimicrobial agents at the early stages of the infection represent an example of the effects of this research. Mathematical analysis is most often used also for case-control studies. The case-control design is well adapted to the identification of rare outcomes, such as infection with resistant microorganisms. In literature we can also find Cohort analysis to determine the incidence of disease or antibiotic resistance in different studied groups [2, 4, 5]. Additionally, the associations between antimicrobial exposure and resistance at the bacterial group level are investigated by using a correlation coefficient based on aggregate data as the measure of association [4, 5]. Another approach, meta-analysis, is a type of systematic survey that applies statistical techniques to include the different research results and arrive at a single quantitative summary (a weighted average) [6].

In recent years, epidemiologists noticed a rapid increase of resistance among pathogenic bacteria species [1, 2, 4, 5, 10]. Uropathogenic *Escherichia coli* strains (UPEC) belong to this group so they are frequently analyzed bacterial species. These bacteria are widespread in the hospital environment and possess mobile genetic elements which take part in the spreading of virulence factors and antibiotic resistance [8]. The most likely cause of acquiring resistance is the inappropriate use of antibiotics to treat bacterial infections [10]. This process becomes increasingly problematic due to the emergence of resistance to several antimicrobial agents, including extended-spectrum cephalosporins, fluoroquinolones, and aminoglycosides [8, 11]. Recent reports show that quinolones are prescribed in nearly 50% of all outpatient urinary tract infection (UTI), although current guidelines recommend against their use as a first-line treatment for uncomplicated UTIs. As a result, the spread of quinolone-resistant pathogens, particularly *E. coli* are on the rise globally [12]. It is important to determine the pathogen resistance and identify the appropriate antibiotic. Such a procedure enables the patient to recover quickly and, in most cases, it does not result in chronic diseases. Susceptibility testing can be done in a number of ways, but the simplest one is to use the method of radial diffusion [11].

Some studies especially emphasize the role of antibiotic consumption in the development of antimicrobial resistance in bacteria [4]. It is well known that the use of antibiotics can lead to the emergence of bacterial resistance [6]. The reasons can be gene mutations, horizontal gene transfer and recombination or changes in the regulation of gene expression, which can influence the construction of the bacterial wall or produce antibacterial proteins. The development of the population of antibiotic-resistant bacteria and their worldwide distribution are the consequence of long years of selective pressure on the grounds of antibiotics underuse, overuse, as well as misuse [13].

Certain antibiotic classes induce resistance more often than others due to the mechanism of action against bacteria. Antibiotic resistance generally arises through mechanisms such as horizontal gene transfer and selection of naturally occurring mutants [4–6, 13]. Cross resistance is a commonly known phenomenon causing resistance to a similarly acting substance. For example, a lot of *E. coli* strains resistant to tetracycline are also insensitive to fluoroquinolones [11]. The mechanism of cross resistance is extremely complicated and unknown, but the most probable cause of this phenomenon would be that chemically dissimilar agents may interfere with the same metabolic pathways. Another explanation is that resistance in a specific case would arise by mutation at one or a few genetic loci, and these mutations result in several phenotypic changes related to origin but are diverse in their effect [14].

Antibiotic resistance patterns are generated for practical reasons. Any drug resistance analyses are essential to discovering the correlations between antibiotics and pathogens and therapy [5]. Analyses of bacterial sensitivity to antibiotics are important for determining the proper dose of antibiotic applied during infection. There are many techniques for differentiating bacteria. Presently, genotyping as PFGE, MLST or PCR based on repeated sequences are the most popular [12, 15–20]. Also the drug resistance patterns can be a potential method for the epidemiological differentiation of bacterial strains which are difficult to distinguish. The profiles of antibiotic sensitivity can be linked with genetic, chemical and/or mathematical analysis. This interdisciplinary approach can deliver a lot of information about the mechanisms of bacterial resistance to antibiotics in their environment [21]. In our previous publication we demonstrated, according to mathematical analysis by Kohonen networks, that clustering of UTI bacteria may depend on resistance/sensitive patterns [8]. In the presented study, the same collection of *E. coli* strains were tested for their drug-resistance profiles of 37 antibiotics. First, the obtained IR bacterial spectra correlated with elaborated artificial neural network were used for the identification of MDR strains from the collection. Next, agglomeration according to Ward’s was used as a tool for deep clonal differentiation of *E.coli* strains. We also compared the strains between each other based on the 1 mm coincidences of diameter zones of inhibited growth by particular antibiotics. Finally, we present the potential method for creating a model for a new look at antibiotic selection during therapy. We use Cohen’s kappa for the observation of specific relationships between antibiotics from the same class.

## Materials and methods

### Bacterial strains

In this study we used a collection of 107 clinical *E. coli* strains isolated from the urine of patients in different wards of military teaching hospital no. 2, medical university of Łódź, Poland in the years 2005–2007. Clinical *E. coli* strains were collected based on the presence of >10^4^ CFU/ml of bacteria in urine. *E. coli* ATCC 25922 was used as a control during an antimicrobial disc diffusion test. The strains were previously characterized based on the presence of virulence factor genes, phylogenetic groups, TRS profiles and drug resistance for 18 antibacterial agents [12]. This collection was later redefined according to the new phylotyping Clermont’s protocol [22], presence of virulence factors according to Adamus-Białek et al. protocol [12] and Muller et al. protocol [23], and TRS-PCR profiles were performed as it was mentioned above [12] (Kubiak-Szeligowska, A., Majchrzak, M., Bartnicka M., 2013, data not shown). The analyzed virulence factor genes were: *papC*, s*faD*/*sfaE, cnf1, usp, hlyA, fimG*/*fimH*.

### Antibiotic susceptibility testing

Antimicrobial susceptibility testing of bacteria was done using the disc diffusion method with commercial discs (Oxoid, Wesel, Germany), according to the guidelines of EUCAST 2015. A standardized inoculum of bacteria (0.5 McFarland standard) were swabbed onto the surface of Mueller–Hinton agar plates (Graso). Filter paper discs impregnated with antimicrobial agents were placed on the agar surface. After 18 ± 2 h of incubation at 35 °C, the diameter of the inhibition zone around each disc was measured, and these measurements were compared with the EUCAST disc diffusion tables. Bacterial isolates were determined to be sensitive (S), intermediate (I), or resistant (R) to the antimicrobial agents tested.

The isolates were tested against 37 antimicrobials: amikacin, amoxicillin, amoxicillin.clavulanate, ampicillin, ampicillin + sulbactam, aztreonam, cefadroxil, cefalexin, cefepime, cefixime, cefotaxime, cefoxitin, ceftazidime, ceftibuten, ceftriaxone, cefuroxime, chloramphenicol, ciprofloxacin, doripenem, ertapenem, fosfomycin, gentamicin, imipenem, levofloxacin, mecillinam, meropenem, moxifloxacin, nalidixic.acid, netilmicin, norfloxacin, ofloxacin, piperacylline, piperacillin + tazobactam, ticarcillin, ticarcillin + clavulanic acid, tigecycline, tobramycin. The analysis was repeated three times for ten randomly selected strains.

### Chemical analysis

The bacterial IR spectra were measured using a “Spectrum 400” spectrometer (Perkin Elmer) in attenuated total reflectance technique (ATR). Detailed conditions of bacterial culture and measurement of infrared spectra were described in previous studies [24]. Briefly, bacteria were grown on LB medium at 37 °C for 24 h. After this time the IR spectrum of a single bacterial colony was measured in the range 4000−750 cm^−1^ with an accuracy of 1 cm^−1^, at a constant temperature and air humidity. For each strain 20 infrared spectra were measured. Then, the first derivatives of absorbance were calculated using five-point stencil and the derivatives were scaled to the range {0–1}.

### Mathematical analysis

The results obtained from drug resistance analysis of the studied strains were used for different mathematical methods. MS Excel and Statistica 10 applications were used for data processing of IR spectra and the design of artificial neural networks. The Euclidean distances between all pairs of strains were calculated and then Ward’s hierarchical clustering method was applied. Bacterial differentiation was performed based on the diameter zone (mm) of inhibited growth on a plate with antibiotics discs. Also, the distributions of inhibition zone diameters for antibiotics were analyzed. Additionally, the relationships between antibiotic effects on the bacteria (resistance, sensitivity) were analyzed using Cohen’s kappa measure of agreement. The correlation with *p*-value < 0.05 was statistical significant. R package irr [(Matthias Gamer, Jim Lemon and Ian Fellows Puspendra Singh < puspendra.pusp22@gmail.com> (2012). irr: Various coefficients of interrater reliability and agreement. R package version 0.84)] was used to calculate Cohen’s kappa.

## Results and discussion

### Application of IR spectrometry and artificial neural network for the identification of MDR strains

**Fig. 1:**
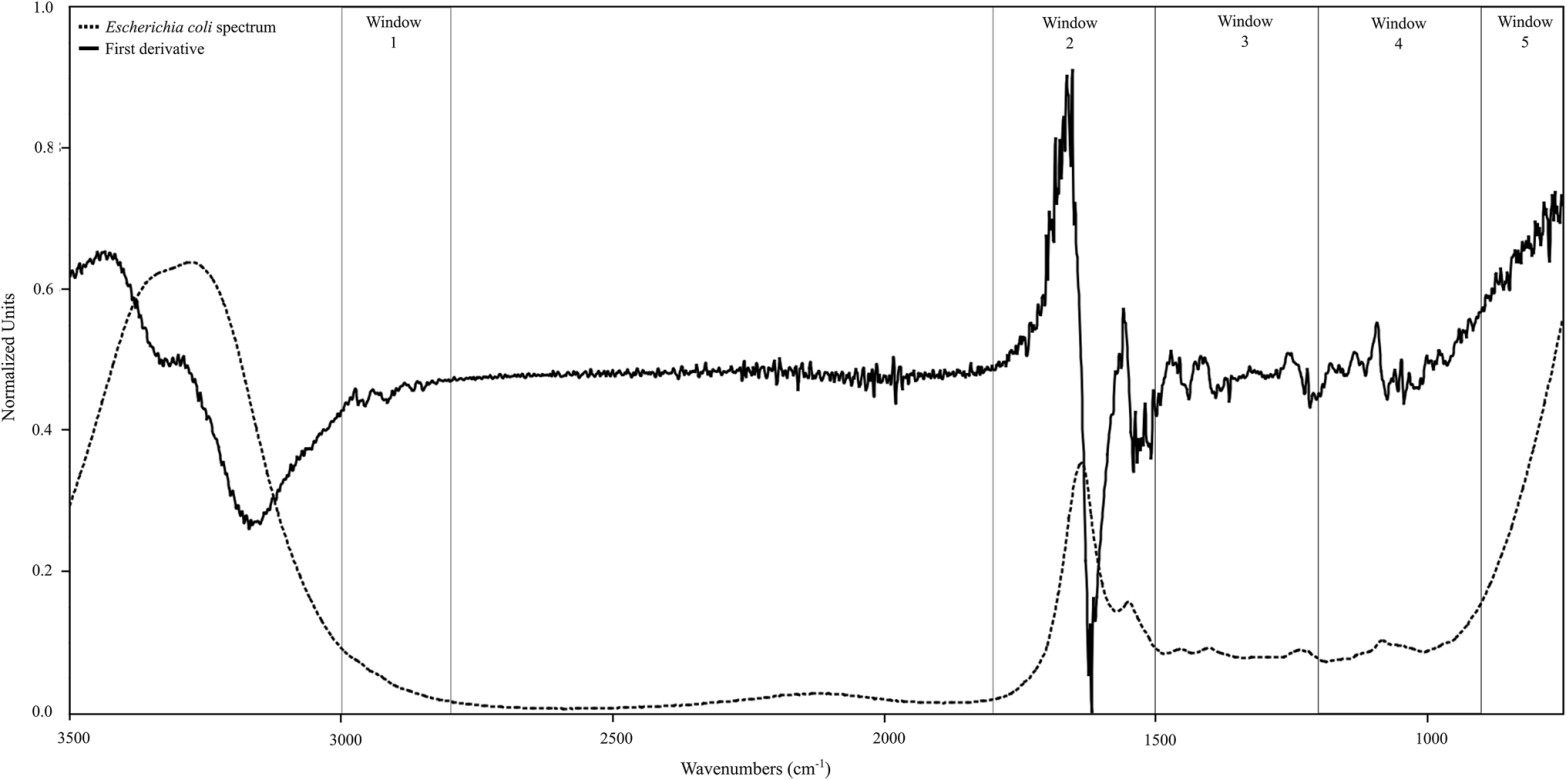
The example of infrared spectrum of uropathogenic *E. coli* strain (No. 41); windows *1*—lipids, windows *2*—proteins, windows *3*—nucleic acids, windows *4*—carbohydrates, windows *5*—fingerprint region

Antimicrobial susceptibility testing of 107 *E. coli* strains for 37 antibiotics was the starting point for further analysis. Based on the disc-diffusion test we identified 41% MDR strains of the total collection of *E. coli* strains. The diverse and valuable approach in the analysis of drug resistance turned out to be IR analysis of bacterial spectra in correlation with artificial neural network. It was shown, that this chemical analysis can also detect these MDR strains. The first derivatives of spectra were used for learning the artificial neural networks (Fig. 1). The IR spectra collection was divided randomly into 3 subgroups: 70% of the spectra were included in the learning group, 15% of the spectra were included in the testing group, 15% of the spectra were included in the validation group. Evaluation of the quality of artificial neural networks was performed on the basis of an error made in the validation group. The best of the neural network recognized properly 92.13% spectra of the bacterial strains in the validation group (Table 1). A similar analysis were published previously. The data of IR bacterial spectra were analyzed in correlation with the gene *papC* [25] and susceptibility for cephalotin [24]. We used the same collection of *E. coli* strains. This method confirmed again its usefulness for the differentiation of bacterial strains. The wide spectrum of organic compounds seen by IR and interpreted by artificial neural network is a great, useful tool for quick screening of clinical bacteria, which can be dangerous and important for epidemiology risk. The results can be obtained the next day and the diagnostics do not require the preparation of the time-consuming disc diffusion test. It can be a new convenient approach for identifying specific properties of other bacterial species. Muhamadali et al. [26] presented similar studies on *Campylobacter* sp. They obtained clear bacterial differentiation using Fourier transform infrared (FT-IR) and Raman spectroscopies, together with matrix-assisted laser desorption ionisation-time of flight-mass spectrometry (MALDI-TOF-MS), as physicochemical approaches for generating biochemical fingerprints. Also Piras et al. [27] used the same technique for bacterial differentiation based on their resistance to enrofloxacin. They also discovered differentially expressed proteins principally involved in antibiotic resistance and linked to oxidative stress response, to DNA protection and to membrane permeability. Elsewhere, Gurbanov et al. [28] used FT-IR spectra for the differentiation of bacteria based on their resistance to heavy metals. We believe that IR bacterial spectra also has great potential in epidemiological differentiation of many species and their features. The broad metabolic and genetic analysis provides new information about bacteria and can even be helpful for developing vaccines against UTI [29]. It should be remembered that bacterial resistance is commonly related to other bacterial properties and mechanisms not directly associated with drug resistance. Understanding the molecular level of those mechanisms is highly important to monitor multi-resistant strains and to expand new therapeutic strategies [30].

**Table 1:** The characteristics of artificial neural networks that recognize *E. coli* MDR strains

No.	Network architecture	Error in learning group (%)	Error in testing group (%)	Error in validation group (%)	Learning algorithm	Error function	Activation function in hiden layer	Activation function in output layer
1	50-8-2	3.54	8.92	7.87	BFGS 156	SOS	Exp	Linear
2	50-9-2	3.2	9.19	8.92	BFGS 158	SOS	Exp	Linear
3	50-10-2	3.66	7.87	8.92	BFGS 123	SOS	Exp	Tanh
4	50-9-2	4.22	9.45	9.19	BFGS 114	SOS	Exp	Linear
5	50-10-2	4.95	8.4	9.45	BFGS 112	SOS	Exp	Tanh
6	50-8-2	6.86	9.45	9.71	BFGS 156	SOS	Exp	Exp
7	50-9-2	5.91	10.24	9.71	BFGS 136	SOS	Exp	Exp
8	50-9-2	4.44	8.66	9.71	BFGS 102	SOS	Exp	Linear
9	50-10-2	3.6	7.61	9.97	BFGS 128	SOS	Exp	Linear

*BFGS* broyden–fletcher–goldfarb–shanno algorithm, *Exp* exponential function, *Tanh* hyperbolic tangent function, *SOS* sum of square

### Bacterial differentiation based on diameter zone of drug resistance

**Fig. 2:**
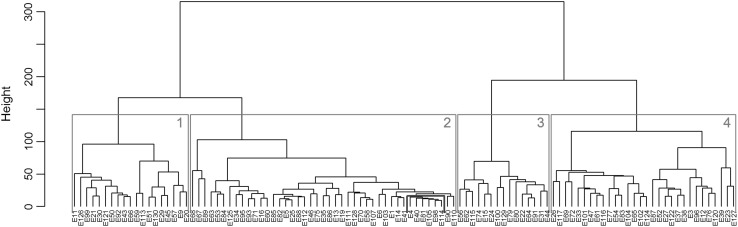
The differentiation of the *E. coli* strains based on the inhibition zones diameters to particular antibiotics according to Ward’s agglomeration method

The reactions of 107 *E. coli* strains to 37 antibiotics were analyzed by Ward’s hierarchical clustering method. Strains were differentiated based on the diameter zone values (mm) of inhibited growth on a plate with antibiotic discs (Fig. 2). The Y axis presents values of the similarity level between bacterial strains. The analysis showed that all strains present different profiles of reaction to antibiotics. However, some strains were grouped based on similarity to each other. The four main clusters were identified in dendrogram. Further, this kind of differentiation revealed a correlation with some kind of bacterial features identified previously [12]. Strains were analyzed based on the virulence factors (VFs) genes specific for uropathogenic *E. coli* strains (*papC*, s*faD*/*sfaE, cnf1, usp, hlyA*) and *fimG*/*fimH* gene specific for the majority of *E. coli* isolates. The studied collection of the bacteria was divided into two groups: with and without VFs genes. Strains with virulence factors possessed at least one uropathogenic VFs gene and *fimG*/*fimH* in contrast to the second group of strains lacking UPEC-specific virulence factors. The analysis of the dendrogram is presented in Table 2, in detail. The bacterial strains with the virulence factor genes are rarely present in clusters I and III. Additionally, cluster III represents MDR strains. In contrast, clusters II and IV represent most strains with the virulence factor genes (approx. 65% of the strains from these clusters) and they are characterized by a lower level of drug resistance. It is worth noting that relatively low coefficients of variation between strains in cluster II suggest high similarity of the reaction of these strains to all antibiotics. Furthermore, 67% of these strains possess analyzed virulence factors. It means, that cluster II represents the most numerous and the most homogeneous group of *E. coli* strains. Additionally, among this cluster strains no.: E4, E40, E81, E98, E105 and E114 are very similar. They were sensitive to 27 of the same antibiotics and their reaction to those antibiotics was almost identical. A similar correlation was observed by CGG-PCR method presented in the previous study. It should be noted, that this is further proof that drug resistance is strongly correlated with the virulence factors of *E. coli* strains [12]. It is worth adding that the dependency between the gender of the host and bacterial resistance have been presented in the literature [31–33], but this correlation was not observed in our study, which may be the result of the fact that the number of strains obtained from male is not representative (25% of bacterial collection, data not shown). The relationship between bacterial resistance and other features are often described in literature. For example, advanced molecular studies reveal the importance of quorum sensing [30] and oxidative stress response [27] to bacterial resistance to antibiotics.

**Table 2:** The characteristics of the dendrogram (Fig. 2)

Cluster	No. of strains	All strains restistant to antibiotics (class)	All strains sensitive to	% of strains with virulence factors genes
1	19	Nalidixic acid (quinolones) close to tigecycline (others)	Mecillinam (penicillins); ampicillin.sulbactam (penicillin comb.); Cefadroxil, ceftriaxone (cephalosporins 3); Meropenem (carbapenems)	21
2	43	Tigecycline (others)	Mecillinam (penicillins); ampicillin.sulbactam (penicillin comb.); Meropenem (carbapenems); Cefalexin (cephalosporins 1); cefadroxil (cephalosporins 3)	67
3	15	Amoxicillin, ampicillin, piperacylline, ticarcillin (penicillins); Ticarcillin.clavulanic.acid (penicillin comb.); Ciprofloxacin, levofloxacin, moxifloxacin, nalidixic.acid, norfloxacin, ofloxacin, (quinolones); Tigecycline (others)	Doripenem, meropenem (carbapenems)	13
4	30	Tigecycline (others); close to: piperacylline (penicillins); Ticarcillin.clavulanic.acid (penicillin combinations)	Meropenem, close to ertapenem (carbapenems)	63

Furthermore, we have differentiated these uropathogenic *E. coli* strains based on the coincidence up to 1 mm of the identified diameter zone of inhibited growth by particular antibiotics. The 1 mm was tagged as a critical value of the bacterial reaction to the antibiotic on the plate. All drug resistance profiles of the strains were compared to each other which gave 5671 combinations. The level of bacterial coincidences based on diameter zones of inhibited growth was presented in Table 3. Only five pairs of strains had no coincidence up to 1 mm of diameter zones of any antibiotics. Also, the coincidence was observed for one antibiotic in the case of 50 paired strains representing only 1% of all combinations. Most of the similarities between drug resistance profiles occurred for 13–30% of antibiotics. The coincidences for more antibiotics were increasingly rare. The highest similarity was observed only for one pair of strains. They had coincidence up to 1 mm of diameter zones of 27 antibiotics (which represents 73% of used antibiotics). To conclude, these methods of drug resistance analysis could be alternative methods for bacterial differentiation. They present very deep differentiation of the strains, but it depends on the cut of value of the similarity level. These methods of differentiation may also be used as a potential tool for the epidemiological investigation of infection paths. Strain differentiation is needed to determine clonal transmission in disease sources, to confirm cross-infection in healthcare settings, or to discover evolutionary diversity among bacteria. Nowadays, the most frequently used method for deep differentiation of bacterial strains is genotyping. In the previous study we also proposed a new method for clonal genotyping of the same bacterial collection [12]. The (CGG)_4_ sequence used in primers for PCR reaction proved to be a great tool for the differentiation of bacteria based on their pathogenic properties. Also other repeated sequences or specific genetic regions are adapted for genotyping e.g. ERIC [12, 34, 35], REP, RAPD, BOX, [15, 36]. The more frequently used techniques involve multilocus sequence typing (MLST), pulsed-field gel electrophoresis (PFGE), restriction fragment length polymorphism (RFLP) studies [16–20]. An old method of differentiation concerns phenotyping as enzyme profiles of bacterial strains [37–40]. Multilocus enzyme electrophoresis (MLEE) could be compared to the analysis of antibiotic zones of inhibition. Resistance to beta-lactams is mainly due to the production of enzymes—betalactamases, which might reveal some correlations between these analyses.

**Table 3:** The coincidence up to 1 mm of growth inhibition zones between drug resistance profiles of each pair of all *E. coli* strains

No. (%) of antibiotics		No. (%) of paired strains with 1 mm coincidence	
0	(0)	5	(0.1)
1	(2.7)	50	(1)
2	(5.41)	139	(2.5)
3	(8.11)	241	(4.3)
4	(10.8)	324	(5.7)
5	(13.5)	426	(7.5)
6	(16.2)	503	(8.9)
7	(18.9)	526	(9.3)
8	(21.6)	536	(9.5)
9	(24.3)	518	(9.1)
10	(27)	496	(8.8)
11	(29.7)	449	(7.9)
12	(32.4)	341	(6)
13	(35.1)	302	(5.3)
14	(37.8)	207	(3.7)
15	(40.5)	162	(2.9)
16	(43.2)	115	(2)
17	(45.9)	94	(1.7)
18	(48.6)	78	(1.4)
19	(51.4)	60	(1.1)
20	(54.1)	47	(0.8)
21	(56.8)	29	(0.5)
22	(59.5)	9	(0.2)
23	(62.2)	6	(0.1)
24	(64.9)	3	(0.1)
25	(67.6)	3	(0.1)
26	(70.3)	1	(0.02)
27	(73)	1	(0.02)
28 ≤ 37	(74 ≤ 100)	0	(0)

### Behavior groups of *E. coli* based on drug resistance profiles

**Fig. 3:**
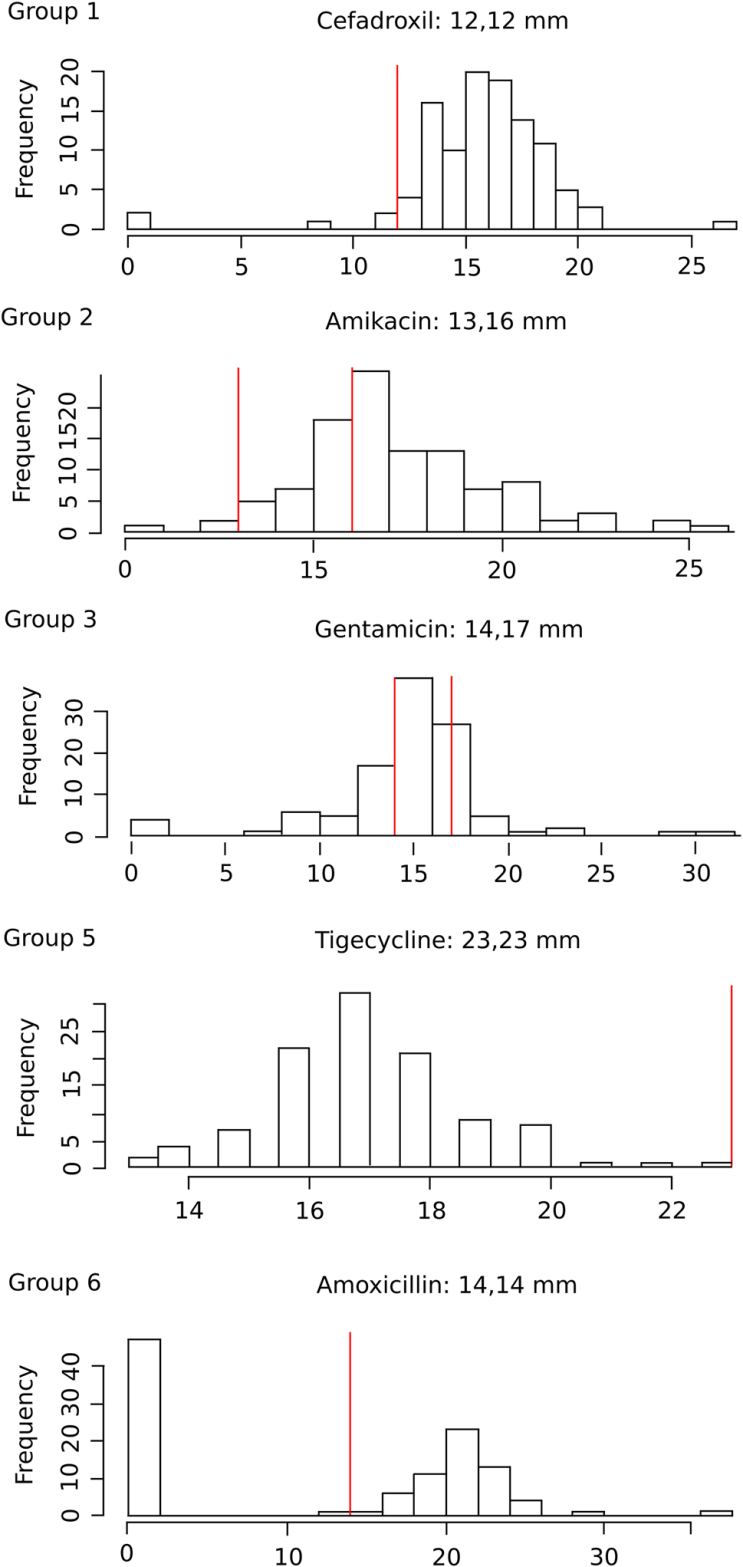
The examples of inhibition zone distributions among studied *E. coli* strains with an appropriate disc potency. Each example represents an identified bacterial behavior group: Sensitive (**1**), Coming intermediate (**2**), Intermediate (**3**), Resistant (**5**), Diverse (**6**) described in Table 1. The behavior groups were designed based on the highest peak of strain distribution in the zones of sensitivity (**1**), intermediate (**3**), resistance (**5**), on the border between the zones (**2, 4**). Diverse group (**6**) represents more than one identified peak of frequency of strains

The analyses were performed based on the distribution of diameter zones of the growth inhibition of all strains for the particular antibiotics. The results were compared to the similar data presented by EUCAST [1, 9]. This source is often used by other scientists to compare their obtained results to the antibiotic resistance of reference strains published by EUCAST or CLSI. These organizations are created to control and estimate the clinical breakpoint changes and their impact on surveillance of antimicrobial resistance [7, 41–45]. The reaction to antibiotics of the studied *E. coli* strains showed different population stages of sensitivity. The behavioristic examples of the population in response to the antibiotic are presented in Fig. 3. Table 4 shows detailed data, where the six groups with different bacterial behavior are designed in a logical way. The frontier zones of sensitivity and resistance represent a cut of value of behavior groups. The highest peak of strains distribution (Fig. 3) classifies the bacterial population to the corresponding behavior group. In the studied collection only five groups were observed. Three homogeneous groups represent “Sensitive”, “Intermediate”, “Resistant” bacterial behavior to antibiotics. Only one antibiotic (tigecycline) classified all strains in the “Resistant” group. Two groups of bacterial behavior were named as changing: “Coming intermediate” and “Coming resistant” based on the highest number of strains on the edge of the zones of sensitivity and resistance. There was no identified antibiotic which classified strains into the “Coming resistant” group. One group of behavior was identified as heterogeneous—“Diverse”. These groups characterize the bacterial population as heterogenic based on more than one highest peak of strains frequency in response to the antibiotic.

**Table 4:** The bacterial behavior groups to particular antibiotics characterized for the collection of reference *E. coli* strains (EUCAST) and for the studied *E. coli* strains based on the pattern in Fig. 3

Group	Bacterial behavior	Antibiotics (reference *E. coli*)	Antibiotics (studied *E. coli*)
1	Sensitive	Amoxicillin, amikacin, **cefadroxil, cefalexin, cefepime, cefixime, cefotaxime**, ceftazidime, **ceftibuten, ceftriaxone**, chloramphenicol, **doripenem, ertapenem**, Imipenem, **mecillinam, meropenem**, moxifloxacin, norfloxacin	Ampicillin.sulbactam, **cefadroxil, cefalexin, cefepime, cefixime, cefotaxime, ceftibuten, ceftriaxone**, cefuroxime, **doripenem, ertapenem, mecillinam, meropenem**
2	Coming intermediate	**Amoxicillin.sulbactam, aztreonam**, cefoxitin, netilmycin, **piperacylline.tazobactam**, tobramycin	Amikacin, **amoxicillin.sulbactam, aztreonam**, ceftazidime, imipenem, **piperacillin.tazobactam**
3	Intermediate	**Ticarcillin.clavulanate**, tigecycline	Cefoxitin, gentamicin, netilmicin, **ticarcillin.clavulanate**, tobramycin
4	Coming resistant	none	none
5	Resistant	none	Tigecycline
6	Diverse	**Ampicillin**, ampicillin.sulbactam, cefuroxime, **ciprofloxacin**, gentamycin, **levofloxacin, nalidixic.acid, ofloxacin, piperacylline, ticarcillin**	Amoxicillin, **ampicillin**, chloramphenicol, **ciprofloxacin, levofloxacin**, moxifloxacin, **nalidixic.acid**, norfloxacin, **ofloxacin, piperacylline, ticarcillin**

Bolded text similarities between strains collections

The analysis of the data from Table 4 led us to conclude that antibiotics (tigecycline) from group 5 (resistant) cannot be used in the therapy of UTI caused by *E. coli*. This is strong evidence that the whole population is resistant to this antibiotic. Group 3 and 6 deserve special attention. The use of antibiotics from these groups is quite hazardous. It is strongly possible that using these antibiotics in UTI therapy will lead the population of *E. coli* to acquire resistance in the near future. Antibiotics from Group 3 belong mainly to aminoglycosides. Quinolones seem to be the most risky antibiotics, because they belong only to Group 6. Strains react very differently to these antibiotics. Also beta-lactam antibiotics from Group 6, which have been used for many years in medicine (amoxicillin, ampicillin), generated a large group of resistant strains of *E. coli* population. It would be good to avoid these antibiotics until the bacterial population returns to the 1st group. The application of antibiotics from the 2nd group should also be done with caution and not used as often as antibiotics from the 1st group. The antibiotics from Group 1 seem to be the strongest, which is obvious because they belong to the new beta-lactam antibiotics. The comparison of these two collections of *E. coli* strains (EUCAST and studied) revealed many differences between behavior groups. These differences result from the shift of the antibiotics into the higher group of studied bacteria, which means increasing resistance to the antibiotic. The situation concerns amikacin, ceftazidime, imipenem, cefoxitin, netilmicin, tobramycin, tigecycline, amoxicillin, chloramphenicol, moxifloxacin, norfloxacin. It means that in Poland, these antibiotics are more popular than in the rest of Europe. The differences in the resistance could be due to variation in the policy of antibiotics use and the pattern of antibiotic prescribing in the local community. A similar comparative analyses were presented in other articles. Konca et al. [46] presented antimicrobial resistance patterns depending on the region, time and other features, which can indicate the specific trends of antibiotic susceptibility patterns of clinical bacteria.

### Antibiotic associations by Cohen’s kappa

**Fig. 4:**
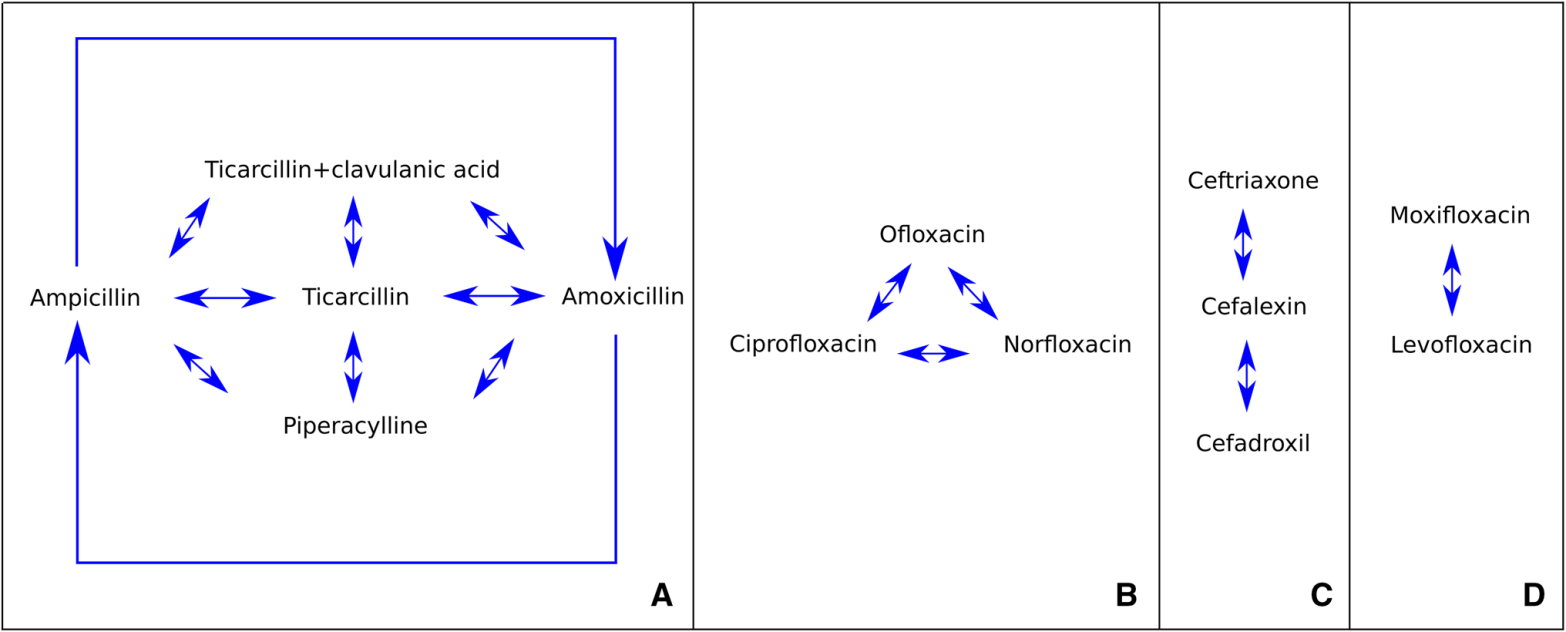
The synergistic effect of antibiotics detected by Cohen’s kappa correlation; ​A—penicillins, B—second generation of fluoroquinolones, C—first and third generation of caphalosporins, D—fourth generation of fluoroquinolones. The correlation is statistically significant (*P* < 0.05, Wald test)

The resistance profiles of studied *E. coli* strains were used for further mathematical analysis. Similarities between the bacterial reaction to various antibiotics were observed. Cohen’s kappa led to indicate specific antibiotic associations. The results discovered two different but important relationships between antibiotics—antibiotics present the same effect on the studied bacterial collection (synergistic effect) (Fig. 4) and antibiotics from the same class with opposite reactions (contrary effect) (Table 5). To better understand this, there were no examples of inconsistency for the same drug. This does not mean that all strains were resistant or sensitive to the antibiotic, but the reaction to the antibiotic of all studied bacterial strains was statistically significant. We analyzed only one collection of the bacterial population, and Cohen kappa analysis shows how the entire set of strains reacts to the particular antibiotic. We could see which antibiotics have a similar (synergistic) or different (antagonistic) effect on the studied bacterial population in this way. The inconsistency of the same antibiotic can be observed in the case of the analysis for different bacterial collections (two different bacterial groups). The biggest group of the compatible antibiotics represent penicillins (A), such as ampicillin, amoxicillin and ticarcillin have the biggest association, and also ticarcillin with clavulanic acid and piperacylline in the same cluster (Fig. 4). The next cluster forms second generation of fluoroquinolones: ofloxacin, ciprofloxacin and norfloxacin (B—Fig. 4), associated with each other. The smaller group (C—Fig. 4) represents the correlation between ceftriaxone (third generation of caphalosporins) and cephalexin (first generation of cephalosporin), and between cephalexin and cefadroxil (first generation of cephalosporin). The correlation between fourth generation of fluoroquinolones was also observed: moxifloxacin and levofloxacin (D). It can constitute the suggestion that they can be used alternatively, because they revealed the same effect on the bacterial population. Therefore, the antibiotic that causes the least side effects can be selected for therapy e.g. ampicillin or ticarcillin instead of amoxicillin. On the other hand, antibiotics from the same cluster should not be used in the case of reinfections. It is highly probable that clinical strains will possess resistance to all antibiotics from the same cluster presented in Fig. 4. This correlation can also be linked to the cross-resistance phenomenon in bacterial population. A similar analysis was presented by Obolski et al. [47] using another mathematical model. They observed a few different co-occurrence patterns of drug resistance of *E. coli* strains. The co-occurrence patterns most similar to our results occurred mainly between some penicillins and penicillin combinations.

**Table 5:** The contrary effect of antibiotics from the same class detected by Cohen’s kappa

Class	Antibiotic	Alterable with	
Penicillins	Mecillinam	Each other	Amoxicillin.clavulanate, ampicillin_sulbactam, piperacillin.tazobactam
	Amoxicillin	Mecillinam	
	Ampicillin		
	Ticarcillin		
	Piperacylline		All penicillin comb
Penicillin combinations	Amoxicillin.Clavulanate	Each other	Each penicillins
	Ampicillin_sulbactam		
	Piperacillin.tazobactam		
	Ticarcillin.clavulanic.acid		Piperacylline
Cephalosporins 2	Cefoxitin	Each other	
	Cefuroxime		
Cephalosporins 3	Cefixime	All cephalosporins 3	
	Cefotaxime		
	Ceftazidime	Cefixime, cefotaxime	
	Ceftibuten	Cefixime, cefotaxime, ceftriaxone	
	Ceftriaxone	Cefixime, cefotaxime, ceftibuten	
Quinolones	Ciprofloxacin	Levofloxacin, moxifloxacin	
	Levofloxacin	Ciprofloxacin, norfloxacin, ofloxacin	
	Moxifloxacin	Ciprofloxacin, norfloxacin, ofloxacin	
	Norfloxacin	Levofloxacin, moxifloxacin	
	Ofloxacin	Levofloxacin, moxifloxacin	
Aminoglycosides	Amikacin	Gentamicin, tobramycin	
	Gentamicin	Amikacin, tobramycin	
	Tobramycin	Amikacin, gentamicin	
	Netilmicin	*Unalterable*	
Others	Chloramphenicol	Fosfomycin	
	Fosfomycin	Chloramphenicol	
	Tigecycline	*Unalterable*	

The correlation is statistically significant (P < 0.05, Wald test)

The statistically significant inverse dependence was also observed in the same class of antibiotics (Table 5). This correlation was discovered between penicillins, penicillin combinations, second- and third-generation cephalosporins, quinolones, aminoglycosides and others. The biggest group of antibiotics with a contrary effect on bacteria is represented by penicillin combinations. Also, they may be converted with all penicillins. There is a strong potential among them, because there is a high applicability of these antibiotics in the case of a resistant strain to one of them, alterable with each other. We also observed that the antibiotic has higher application potential if it belongs to a higher generation, for example quinolones. Levofloxacin and moxifloxacin belong to a later generation in comparison to ciprofloxacin, norfloxacin and ofloxacin, so they characterize an opposite effect on the bacterial population. This correlation can be a helpful tip to choose another antibiotic of the same class in the case of reinfection, routine or quick decision-making about antibiotic therapy.

A similar comparative analysis was performed in our previous study, where we presented the associations between antibiotics [8]. The presented correlations between antibiotics on the Kohonen maps identify the direction of emergence of drug resistance with high probability. The correlation between antibiotic co-resistance was also presented by Pathak et al. [21] and Obolski et al. [47], where the authors indicate that resistance to antibiotic can be connected with others. Also, Boonten et al. [4] presented the combination of the obtained results with mathematical modeling to determine the quantitative effects of individual infection control measures. It should be emphasized that drug resistance of bacteria are connected with many different features of bacteria, their host and antibiotic policy.

## Summary

We demonstrated a detailed and broad approach to the antibiotic resistance of uropathogenic *E. coli* strains. The studied bacterial population make an example of pathogens, which can be analyzed in these ways for a better understanding of their associations with antibiotic therapy. The conclusions were drawn based on the statistically significant results, but they concern only one bacterial population. The state of antibiotic profiles have changed in 10 years. These results should be verified by fresh bacterial collection. However, the results were compared to the drug resistance profile from EUCAST data. We should also remember the potential different pathways of bacterial adaptation to survive in environments with antibiotic. Based on the observed findings we would like to pay special attention to several important aspects:

1. IR spectra with the correlations of artificial neural network may be taken into account for routine and quick diagnostic of MDR strains.
2. Ward’s hierarchical clustering method based on the diameter zone of inhibited growth of bacterial strains could be used for clonal and epidemiological bacterial differentiation.
3. The use of tigecycline should be reconsidered against uropathogenic *E. coli* strains.
4. Fluoroquinolones as well as ampicillin, piperacylline and ticarcillin seem to be the most risky antibiotics, because they represent bacterial behavior belonging only to Group 6 (Diverse).
5. Both Table 3 and Fig. 4 may be the starting point to create a helpful model when deciding on what antibiotic to select against UPEC.

## References

[1] Kahlmeter G (2014). Defining antibiotic resistance-towards international harmonization. Upsala J Med Sci.

[2] Lipsitch M, Bergstrom CT, Levin BR (2000) The epidemiology of antibiotic resistance in hospitals: paradoxes and prescriptions. Proc Natl Acad Sci 97:1938–1943. http://www.ncbi.nlm.nih.gov/pmc/articles/PMC26540/pdf/pq001938.pdf 10.1073/pnas.97.4.1938 PMC26540 10677558

[3] Cameron E, Battle KE, Bhatt S, Weiss DJ, Bisanzio D, Mappin B, Dalrymple U, Hay SI, Smith DL, Griffin JT, Wenger EA, Eckhoff PA, Smith TA, Penny MA, Gething PW (2015). Defining the relationship between infection prevalence and clinical incidence of *Plasmodium falciparum* malaria. Nat Commun.

[4] Bonten MJM, Austin DJ, Lipsitch M (2001). Understanding the spread of antibiotic resistant pathogens in hospitals: mathematical models as tools for control. Clin Infect Dis.

[5] Schechner V, Temkin E, Harbarth S, Carmeli Y, Schwaber MJ (2013). Epidemiological interpretation of studies examining the effect of antibiotic usage on resistance. Clin Microbiol Rev.

[6] Davies J, Davies D (2010). Origins and evolution of antibiotic resistance. Microbiol Mol Biol Rev.

[7] Matuschek E, Brown DFJ, Kahlmeter G (2014). Development of the EUCAST disk diffusion antimicrobial susceptibility testing method and its implementation in routine microbiology laboratories. Clin Microbiol Infect.

[8] Adamus-Bialek W, Zajac E, Parniewski P, Kaca W (2013). Comparison of antibiotic resistance patterns in collections of *Escherichia coli* and *Proteus mirabilis* uropathogenic strains. Mol Biol Rep.

[9] Kahlmeter G (2015). The 2014 Garrod Lecture: EUCASTare we heading towards international agreement?. J Antimicrob Chemother.

[10] Chojecka A, Jakimiak B, Röhm-Rodowald E, Podgórska M (2010). The effect of antibacterial substances on spread resistance of bacteria. Epidemiol Rev.

[11] Hwang TJ, Hooper DC (2014). Association between fluoroquinolone resistance and resistance to other antimicrobial agents among *Escherichia coli* urinary isolates in the outpatient setting: a national cross-sectional study. J Antimicrob Chemother.

[12] Adamus-Bialek W, Wojtasik A, Majchrzak M, Sosnowski M, Parniewski P (2009). (CGG)4-based PCR as a novel tool for discrimination of uropathogenic *Escherichia coli* strains: comparison with enterobacterial repetitive intergenic consensus-PCR. J Clin Microbiol.

[13] Woo PC, To AP, Lau SK, Yuen KY (2003). Facilitation of horizontal transfer of antimicrobial resistance by transformation of antibiotic-induced cell-wall-deficient bacteria. Med Hypotheses.

[14] Kohanski MA, DePristo MA, Collins JJ (2010). Sublethal antibiotic treatment leads to multidrug resistance via radical—induced mutagenesis. Mol Cell.

[15] de la Puente-Redondo VA, del Blanco NG, Gutierrez-Martin CB, García-Pena FJ, Rodriguez Ferri EF (2000) Comparison of different PCR approaches for typing of *Francisella tularensis* strains. J Clin Microbiol 38(3):1016–1022. http://www.ncbi.nlm.nih.gov/pmc/articles/PMC86326/10.1128/jcm.38.3.1016-1022.2000 PMC86326 10698989

[16] Chan KG, Loke MF, Ong BL, Wong YL, Hong KW, Tan KH, Kaur S, Ng HF, Abdul Razak M, Ngeow YF (2015). Multiphasic strain differentiation of a typical mycobacteria from elephant trunk wash. PeerJ.

[17] O’Hara FP, Suaya JA, Ray GT, Baxter R, Brown ML, Mera RM, Close NM, Thomas E, Amrine-Madsen H (2016). spa Typing and multilocus sequence typing show comparable performance in a macroepidemiologic study of *Staphylococcus aureus* in the United States. Microb Drug Resist.

[18] Andersson P, Tong SYC, Bell JM, Turnidge JD, Minim PMG (2012). Typing—a rapid and low cost MLST based typing tool for *Klebsiella pneumoniae*. PLoS ONE.

[19] Nowakiewicz A, Ziółkowska G, Zięba P, Gnat S, Wojtanowicz-Markiewicz K, Trościańczyk A (2016). Coagulase-positive Staphylococcus isolated from wildlife: identification, molecular characterization and evaluation of resistance profiles with focus on a methicillin-resistant strain. Comp Immunol Microbiol Infect Dis.

[20] Larsen MV, Cosentino S, Rasmussen S, Friis C, Hasman H, Marvig RL, Jelsbak L, Sicheritz-Ponten T, Ussery DW, Aarestrup FM, Lund O (2012). Multilocus sequence typing of total-genome-sequenced bacteria. J Clin Microbiol.

[21] Pathak A, Chandran SP, Mahadik K, Macaden R, Stalsby Lundborg C (2013). Frequency and factors associated with carriage of multi-drug resistant commensal *Escherichia coli* among women attending antenatal clinics in Central India. BMC Infect Dis.

[22] Clermont O, Christenson JK, Denamur E, Gordon DM (2013). The Clermont *Escherichia coli* phylotyping method revisited: improvement of specificity and detection of new phylo-groups. Environ Microbiol Rep.

[23] Muller D, Greune L, Heusipp G, Karch H, Fruth A, Tschape H, Schmidt MA (2007). Identification of unconventional intestinal pathogenic *Escherichia coli* isolates expressing intermediate virulence factor profiles by using a novel single-step multiplex PCR. Appl Environ Microbiol.

[24] Lechowicz Ł, Urbaniak M, Adamus-Białek W, Kaca W (2013) The use of infrared spectroscopy and artificial neural networks for detection of uropathogenic *Escherichia coli* strains susceptibility to cephalothin. Acta Biochim Pol 60(4):713–718. http://www.actabp.pl/pdf/4_2013/713.pdf 24432322

[25] Lechowicz Ł, Adamus-Białek W, Kaca W (2013). Attenuated total refectance fourier transform infrared spectroscopy and artificial neural networks applied to differentiate *Escherichia coli* pap+/pap−strains. J Spectrosc.

[26] Muhamadali H, Weaver D, Subaihi A, Al Masoud N, Trivedi DK, Ellis DI, Linton D, Goodacre R (2016). Chicken, beams, and Campylobacter: rapid differentiation of foodborne bacteria via vibrational spectroscopy and MALDI-mass spectrometry. Analyst.

[27] Piras C, Soggiu A, Greco V, Martino PA, Del Chierico F, Putignani L, Urbani A, Nally JE, Bonizzi L, Roncada P (2015). Mechanisms of antibiotic resistance to enrofloxacin in uropathogenic *Escherichia coli* in dog. J Proteomics.

[28] Gurbanov R, Simsek Ozek N, Gozen AG, Severcan F (2015). Quick discrimination of heavy metal resistant bacterial populations using infrared spectroscopy coupled with chemometrics. Anal Chem.

[29] Mobley HLT, Alteri CJ (2015). Development of a vaccine against *Escherichia coli* urinary tract infections. Pathogens.

[30] Piras C, Soggiu A, Bonizzi L, Gaviraghi A, Deriu F, De Martino L, Iovane G, Amoresano A, Roncada P (2012). Comparative proteomics to evaluate multi drug resistance in *Escherichia coli*. Mol BioSyst.

[31] Alos JL, Serrano MG, Gomez-Garces JL, Perianes J (2005). Antibiotic resistance of *Escherichia coli* from community-acquired urinary tract infections in relation to demographic and clinical data. Clin Microbiol Infect.

[32] Sahm DF, Thornsberry C, Mayfield DC, Jones ME, Karlow JA (2001). Multidrug-resistant urinary tract isolates of *Escherichia coli*: prevalence and patient demographics in the United States in 2000. Antimicrob Agents Chemother.

[33] Edlin RS, Shapiro DJ, Hersh AL, Copp HL (2013). Antibiotic resistance patterns of outpatient pediatric urinary tract infections. J Urol.

[34] Ibrahim DR, Dodd CE, Stekel DJ, Ramsden SJ, Hobman JL (2016) Multi drug and extended spectrum beta-lactamase resistant Escherichia coli isolated from a dairy farm. FEMS Microbiol Ecol pii.fiw013. doi:10.1093/femsec/fiw013 10.1093/femsec/fiw013 26850161

[35] Rakotonirina HC, Garin B, Randrianirina F, Richard V, Talarmin A, Arlet G (2013). Molecular characterization of multidrug-resistant extended-spectrum β-lactamase-producing Enterobacteriaceae isolated in Antananarivo, Madagascar. BMC Microbiol.

[36] Aladag MO, Uysal A, Dundar N, Durak Y, Gunes E (2013) Characterization of *Klebsiella pneumoniae* strains isolated from urinary tract infections: detection of ESBL characteristics, antibiotic susceptibility and RAPD genotyping. Pol J Microbiol 62(4):401–409. http://www.pjm.microbiology.pl/archive/vol6242013401.pdf 24730135

[37] Frandsen EV, Wade WG (1996). Differentiation of human Capnocytophaga species by multilocus enzyme electrophoretic analysis and serotyping of immunoglobulin A1 proteases. Microbiology.

[38] Souza V, Rocha M, Valera A, Eguiarte LE (1999) Genetic structure of natural populations of *Escherichia coli* in wild hosts on different continents. Appl Environ Microbiol 65(8):3373–3385. http://www.ncbi.nlm.nih.gov/pmc/articles/PMC91507/pdf/am003373.pdf 10.1128/aem.65.8.3373-3385.1999 PMC91507 10427022

[39] Johnson JR, O’Bryan TT (2000) Improved repetitive-element PCR fingerprinting for resolving pathogenic and nonpathogenic phylogenetic groups within *Escherichia coli*. Clin Diagn Lab Immunol 7(2):265–273. http://www.ncbi.nlm.nih.gov/pmc/articles/PMC95859/pdf/cd000265.pdf 10.1128/cdli.7.2.265-273.2000 PMC95859 10702503

[40] Campos LC, Zahner V, Avelar KE, Alves RM, Pereira DS, Vital BJ, Freitas FS, Salles CA, Karaolis DK (2004). Genetic diversity and antibiotic resistance of clinical and environmental *Vibrio cholerae* suggests that many serogroups are reservoirs of resistance. Epidemiol Infect.

[41] van der Bij K, van Dijk K, Muilwijk J, Thijsen SFT, Notermans DW, de Greeff S, van de Sande-Bruinsma N (2012). Clinical breakpoint changes and their impact on surveillance of antimicrobial resistance in Escherichia coli causing bacteraemia. Clin Microbiol Infect.

[42] Hombach M, Mouttet B, Bloemberg GV (2013). Consequences of revised CLSI and EUCAST guidelines for antibiotic susceptibility patterns of ESBL- and AmpC b-lactamase-producing clinical Enterobacteriaceae isolates. J Antimicrob Chemother.

[43] Cuesta I, Bielza C, Cuenca-Estrella M, Larranaga P, Rodríguez-Tudela JL (2010). Evaluation by data mining techniques of fluconazole breakpoints established by the clinical and laboratory standards institute (CLSI) and comparison with those of the European Committee on antimicrobial susceptibility testing (EUCAST). Antimicrob Agents Chemother.

[44] Bengtsson S, Bjelkenbrant C, Kahlmeter G (2014). Validation of EUCAST zone diameter breakpoints against reference broth microdilution. Clin Microbiol Infect.

[45] Wolfensberger A, Sax H, Weber R (2013). Change of antibiotic susceptibility testing guidelines from CLSI to EUCAST: influence on cumulative hospital antibiograms. PLoS ONE.

[46] Konca C, Tekin M, Uckardes F, Akgun S, Almis H, Bucak IH, Genc Y, Turgut M (2016). An overview of antibacterial resistance patterns of pediatric community-acquired urinary infections. Pediatr Int.

[47] Obolski U, Dellus-Gur E, Stein GY, Hadany L (2016). Antibiotic cross-resistance in the lab and resistance co-occurrence in the clinic: discrepancies and implications in *E.coli*. Infect Genet Evol.

